# Oral Health-Related Quality of Life in Children at a Pediatric Emergency Dental Service During the Start of COVID-19

**DOI:** 10.3390/dj13040171

**Published:** 2025-04-18

**Authors:** Ali Al Ibraheem, Clara Dürsch, Katrin Bekes

**Affiliations:** Department of Paediatric Dentistry, University Clinic of Dentistry, Medical University Vienna, Sensengasse 2A, 1090 Vienna, Austria; ali.alibraheem@meduniwien.ac.at (A.A.I.);

**Keywords:** oral health, dental pain, pediatric dentistry, quality of life, COVID-19

## Abstract

**Background/Objectives:** Oral health-related quality of life (OHRQoL) has become increasingly significant in dentistry. By incorporating patient perspectives through questionnaires alongside objective diagnoses, the understanding of diseases is enhanced. This study examines OHRQoL during the early COVID-19 pandemic in children aged 0–10 who visited the Emergency unit of Pediatric Dentistry at the Medical University of Vienna from March to May 2020, focusing on the impact of COVID-19 restrictions on various social and health aspects. The study aimed to assess OHRQoL in children during the early weeks of the COVID-19 pandemic and correlate it with other health and social conditions. **Materials and Methods:** Children (up to 10 years) and their parents visiting the Emergency Unit of the department completed OHRQoL questionnaires. In children aged 0–6, their parents answered the Early Childhood Oral Health Impact Scale questionnaire (ECOHIS), while 7–10-year-olds completed the Child Perception Questionnaire (CPQ) by themselves. Summary scores and associations between oral and general health subdomains were analyzed. **Results:** Both the ECOHIS and CPQ groups showed high general summary scores, indicating decreased OHRQoL (ECOHIS 12.22 [±8.68] and CPQ 18.36 [±12.35]). The highest scores were in the “oral symptoms” domain, mainly due to “toothache”. Significant correlations were observed between “general health” and “oral health” with the “family section” in the ECOHIS group and between “oral health” and “oral symptoms” with “emotional well-being” in the CPQ group. **Conclusions:** During the pandemic, both age groups experienced decreased OHRQoL. Family background influenced oral health (ECOHIS), while oral symptoms and emotional well-being correlated with oral health (CPQ). Further research is needed to understand pandemic-related factors affecting OHRQoL and develop preventive strategies.

## 1. Introduction

With the beginning of the coronavirus, the quality of life in Austria changed significantly [1]. By October 2020, surveys indicated that 69% of the population found the COVID-19 crisis “very stressful” or “rather stressful”. Mobility restrictions, curfews, and quarantine measurements limited oral health care and particularly dental visits. Non-necessary treatments at the University Dental Clinic in Vienna, Austria, were postponed due to high infection risks and staff shortages, allowing only emergency care for urgent dental treatments such as acute trauma or pain [2]. Initially, dental treatments were limited to emergency services [3]. However, the adequate management and treatment of dental pain are essential in pediatric dentistry, as dental caries is one of the most common problems affecting children, with a prevalence ranging from 30 to 90%. This condition imposes a heavy burden on patients and their families, potentially impacting their quality of life, general health, and well-being [4,5]. In recent years, the concept of oral health-related quality of life (OHRQoL) has become an essential measure for examining the impact of oral conditions on the psychosocial well-being of both children and adults [6,7]. By including the patient’s or parent’s self-perceived oral health and needs in terms of social or psychological impacts, OHRQoL provides a comprehensive assessment of patients’ oral health, complementing traditional diagnostic criteria in clinical practice. This has also led to the development of various questionnaires and evaluation methods to collect data directly from patients, ultimately helping to improve everyday practice [8,9]. One of the widely used instruments to measure OHRQoL is the Child Perception Questionnaire (CPQ), designed for children aged 8–10. For young patients, the Early Childhood Oral Health Impact Scale (ECOHIS) can be applied to investigate the relationship between parental perception of the quality of life of their preschool children and their oral health status. While many studies have investigated quality of life (QoL) under various circumstances, there is a particular need for research on general QoL and oral health-related quality of life (OHRQoL) during COVID-19 and its global impact on both patients and practitioners.

Therefore, this study aims to analyze the oral health-related quality of life (OHRQoL) in children who presented at the Emergency Dental Service of the Department of Pediatric Dentistry, University Dental Clinic, Vienna, Austria at the beginning of the COVID-19 pandemic from March to early May 2020.

## 2. Materials and Methods

The present study was planned and conducted at the Department of Pediatric Dentistry at the University Dental Clinic Vienna, Medical University. A positive ethics vote was obtained (Ethics vote: 1822/2015, 2025/2015). The data collection was initially planned for the first eight weeks of the SARS-CoV-2 pandemic, from March to early May 2020. However, it was delayed and conducted from 3 April to 2 June 2020. All pediatric patients and their legal guardians who presented to the Emergency Unit during this period were informed about the possibility of participating in the study.

After obtaining written consent from the legal guardians (along with verbal consent from the children), the patients or accompanying persons were provided with the questionnaire standardized for their age. After the allocation of a patient ID (pseudonymization), an OHRQoL questionnaire had to be completed. Additionally, the diagnosis and reason for presentation were noted in the patient file.

For children aged 0 to 6 years, the German version of the Early Childhood Oral Health Impact Scale was used, and for those aged 7 to 10 years, the German version of the Child Perceptions Questionnaire, version 8–10, was employed. The ECOHIS-G was filled out by the parents or legal guardians, while the CPQ was completed by the children themselves [10,11].

The ECOHIS, as previously mentioned, is an instrument primarily used for preschool children to assess OHRQoL. It contains 13 questions that are answered by the parents and consists of 2 parts. The first part, focusing on the impacts of oral health-related quality of life (OHRQoL) on the children themselves (“child section”), consists of four domains and a total of nine questions [11,12,13]: (1) oral symptoms (one question), (2) child-related function (four questions), (3) child’s psychology (two questions), and (4) child’s self-image and social interaction (two questions). The second part addresses the impacts of MLQ on the family (“family section”) and includes two domains with a total of four questions: (1) parental distress/suffering (two questions) and (2) family-related function (two questions).

The CPQ consists of 25 items divided into 4 subscales [14]: (1) oral symptoms (5 questions), (2) functional limitations, (3) emotional well-being, and (4) social well-being. Additionally, there are two questions regarding general oral health and overall health, which respondents can rate as “excellent” = 4, “very good” = 3, “good” = 2, “fair” = 1, and “poor” = 0. In these questions, a high value corresponds to an exceptionally good (oral) health status, unlike the rest of the items.

Attending nurses (five nurses in total) collected the data, which were then reviewed by dentists. The researchers conducted data transfer, summarization, and processing. Patients were excluded if they failed to complete the questionnaires correctly or left sections unanswered. Additionally, those with language difficulties were excluded, as full comprehension of the questions was essential.

This study employs a retrospective quantitative design based on questionnaire data. Data from the questionnaires were manually transferred into an Excel spreadsheet and into the statistical analysis software IBM SPSS Statistics Version 27 (IBM Corp., Armonk, NY, USA). Prior to analysis, a Pearson correlation coefficient of 0.30 and a significance level of 0.05 were set in SPSS. This was then cross-checked by a second person involved in the study planning. The diagnosis or reason for presentation of the patients was supplemented from the clinical patient records. If a patient had two relevant diagnoses, both were included in the database.

Nominal measurements (e.g., gender, type of diagnosis) were summarized using frequencies and proportions along with cross tabulations. T-tests for independent samples were calculated to compare interesting proportions between both groups. Analysis of variance (ANOVA) was conducted to assess statistical significance and subsequently examined for validity through correlation analysis. Correlation analysis was performed to analyze the effect of different factors on global oral health, overall well-being, and OHRQoL (ECOHIS scores).

## 3. Results

Out of a total of 103 participants, 4 were excluded due to incorrectly filled out questionnaires, resulting in only 99 children (46% female, 53% male) being included in the study. Subsequently, it was found that the overall population had not been strictly separated according to the age criteria. For organizational reasons, the ECOHIS group (n = 46) included 2 subjects who were older than 6 years, and the CPQ group (n = 53) included 29 subjects whose ages were outside the range of 8–10 years. A particular difficulty arose with the 7-year-olds (n = 11), the age group that fell between the ECOHIS and CPQ subjects. Of them, 9 received the CPQ questionnaire and 2 received the ECOHIS questionnaire. Table 1 and Table 2 summarize the characteristics of the study population.

Regarding the most common problems, as seen in Table 2, the response frequencies of the six most common oral problems among ECOHIS respondents are listed. Accordingly, 26.1% of respondents reported being “hardly” affected by “pain”, while 28.3% reported being “never” affected. Additionally, 19.6% reported experiencing “occasional” pain, and 17.4% reported experiencing “frequent” pain. For more than two-thirds (69.6%) of respondents, “pain” was likewise “never” a problem, and the “absence from kindergarten/daycare/school” due to oral symptoms had “never” occurred in most children (73.9%) so far. “Sleeping problems” mostly also “never” (60.9%) arose.

In the younger age group, measured with the ECOHIS, 95.7% of parents rated the “general health status” of their child as “good”, “very good”, or “excellent”, while 4.4% rated it as “fair” or “poor”. However, only 65.2% rated the “oral health status” as “good” or better, with 34.8% rating it as “fair” or “poor”. As shown in Table 3, no statistically significant association was found between the ECOHIS total score and the “general health status” (ρ = 0.035; *p* = 0.82 > 0.05). The correlation of the “child section” domain with the “general health status” was also found to be non-significant (ρ = 0.18, *p* = 0.24 > 0.05). In contrast, a significant association was observed between the total score of the “family section” domain and the total score of the “general health status” (ρ = 0.31; *p* = 0.03 < 0.05). However, similar to the “general health status”, the association between the “oral health status” and the “family section” was significant (ρ = 0.33; *p* = 0.03 < 0.05), as seen in Table 3.

More than two-thirds (67.4%) of the children presented to the Emergency Unit at the Department of Pediatric Dentistry in Vienna due to caries-related pain. Among the 31 subjects with caries, 11 additionally suffered from swelling/fistula. Thus, 35.5% of the caries patients and 23.9% of all 46 ECOHIS subjects had a fistula as a complication of caries. Table 4 presents the ECOHIS total score as well as the domain total scores in the group of the two most common diagnoses, caries (n = 31), and anterior tooth trauma (n = 9). In the caries subpopulation, the mean ECOHIS total score was 13.77 (±9.885; range 0–42), and the domain total score in the “child section” averaged 7.68 (±7.54; range 0–30). In the “family section”, the domain total score averaged 2.90 (±2.88; range 0–8). Patients with the second most common diagnosis, anterior tooth trauma (n = 9), had an average ECOHIS total score of 7.33 (±3.64; range 0–13) and a domain total score of 2.33 (±3.00; range 0–8) in the “child section”. In the “family section”, the average was 0.22 (±0.67; range 0–2).

Moving on to the CPQ group, the average total score when answering the CPQ was 18.36 (±12.35; range 0–50). The occurrence of “oral symptoms” was rated on average with the highest domain sum score of 6.11 (±4.08; range 0–15). Table 5 summarizes the response frequencies for the six most common oral health-related issues of the CPQ. A total of 24.5% of patients reported experiencing toothache “often”, while 20.8% reported experiencing it “occasionally”. The majority of respondents (30.0%) reported experiencing such pain “once or twice” during the specified period, and 17.0% reported having experienced it “never”.

The first two parts of the CPQ capture “general health status” and “oral health status”. In this study, 96.2% of respondents rated their “general health status” as “good”, “very good”, or “excellent”, while 3.8% rated it as “fair” (none rated it as “poor”). However, only 69.8% rated their “oral health status” as “good” or better, while 30.2% rated it as “fair” or “poor”.

To examine the relationships between “general health status” and the CPQ sum score as well as the individual domains, Spearman’s rank correlation coefficient (ρ) was calculated analogously to the ECOHIS evaluation and tested for significance using the *p*-value (*p*) at a significance level of 0.05 (Table 6). Similarly, the “oral health status” was tested for correlations (Table 6). No significant correlation between the “general health status” and the overall CPQ sum score could be established (ρ = 0.09; *p* = 0.52 > 0.05) (Table 6). The relationship between the “oral health status” and the overall CPQ sum score was also found to be non-significant (ρ = 0.23; *p* = 0.09 > 0.05) (Table 6).

It is noticeable that more than half (50.9%) of the patients showed caries. Of the 27 participants with caries, 4 simultaneously suffered from a fistula or swelling. Thus, 14.8% of the participants had caries, and 7.5% of all participants had a fistula as a complication of caries. Almost a quarter (24.5%) of the respondents had less common problems (“Others”), such as broken fillings or crowns, space maintainer fractures or pressure sores, fractures, hypersensitivity pain, tooth mobility, or teething pain. The most common diagnosis, “caries” (n = 27), is analyzed in more detail regarding the response behavior in the CPQ (Table 7). A decreasing mean value was observed from domain 1 to domain 4 (with their ascending numerical designations). In domain 1, “oral symptoms”, the average sum score was the highest at 5.85 (±4.25; range 0–15). In domain 2, “functional limitations”, the average sum score was 3.41 (±4.12; range 0–12). The scores for emotional and social well-being were lower (3.00 ± 3.44; range 0–12 and 1.04 ± 1.87; range 0–8, respectively).

## 4. Discussion

The study focuses on oral health-related quality of life (OHRQoL) in children aged 0–10 during the early weeks of the COVID-19 pandemic. It aimed to evaluate the impact of COVID-19 restrictions on children’s OHRQoL and its correlation with health conditions. Results showed a decrease in OHRQoL, with the highest impact in the “oral symptoms” domain, primarily due to toothache. Significant correlations were also found between general and oral health with family background (ECOHIS) and between oral health, oral symptoms, and emotional well-being (CPQ).

Families play a crucial role in children’s physical and emotional well-being by providing safety and guidance, which shape their development, health behaviors, and social skills. Children require care that fosters positive emotional health, supports their mental well-being, and helps them develop a strong sense of self. This includes coping with stress, regulating emotions, overcoming fears, and managing disappointments. Parents and caregivers are essential in guiding children through emotional challenges and behavioral management, significantly impacting their overall health and well-being [15,16].

The ECOHIS participants showed a relatively balanced gender distribution, with girls (56%) outnumbering boys (43%). According to the predetermined classification, the age should be between 0 and 6 years. Two of the eleven seven-year-olds were assigned to the ECOHIS instead of the CPQ due to difficulties in self-completing the ECOHIS. Among the CPQ respondents, there was an uneven gender distribution. Almost two-thirds of the included participants were male (62%), while slightly more than one-third were female (38%). When considering the age of the CPQ participants, it ranged from 5 to 14 years at the time of the survey. According to the protocol, participants younger than 8 years old should have answered the ECOHIS instead of the CPQ (ages 8–10), and participants older than 10 years old should have answered the CPQ (ages 11–14) (total n = 29).

The average age of the CPQ participants being 8 years old, with a range of 5–14 years, confirms that most children were in the age group defined by the questionnaire, which is 8–10 years old. This is further supported by the standard deviation (±2.1), which provided little room for variation. It can be concluded that the ECOHIS participants were predominantly older, while the CPQ participants were predominantly younger than the mean age of the age group for which the questionnaire was designed. From this, it can be inferred that only a few patients of the ECOHIS were included at the lower end and few of the CPQ at the upper end of the age range.

The ECOHIS standard deviation (Σ) of 12.22 in this study was significantly higher compared with other studies conducted under normal conditions in various countries: Australia (4.20), Brazil (3.84), China (9.63), Austria (6.10), Mexico (3.20), Peru (8.74), and Thailand (4.15) [17,18,19,20,21,22]. Specifically, the study’s ECOHIS_Σ of 12.22 was notably higher than the 6.12 measured in Austria under normal conditions, indicating a more significant restriction in OHRQoL during the pandemic. This higher deviation is attributed to the study population being from an emergency service rather than routine care. Spanemberg et al. reported that patients in pain services have an eight-fold higher likelihood of experiencing greater OHRQoL impairment compared with those in routine treatment [23]. The range of 0–42 reflected the highly variable perception of OHRQoL, and the ECOHIS_Σ of 12.22 was shifted far to the left in this range, illustrating the relatively low average OHRQoL impairment.

The average poorer OHRQoL of our participants could also be attributed to the emergency situation, as observed in patients treated by the pain service compared with routine treatment. Therefore, the potentially worse OHRQoL of our participants may be justified by the emergency situation [23]. Rauch et al. also found in their survey that patients presenting with pain in German emergency dental care services, in addition to a high prevalence of dental anxiety, exhibited a high level of OHRQoL impairment [24,25]. These findings mitigate the potential influence of the pandemic on the OHRQoL.

In other publications, it has also been demonstrated that “tooth pain” has the strongest impact on individual oral health-related quality of life (1.52 ± 1.30) [17,26,27]. The closely clustered average response values of the remaining ECOHIS questions suggest a similar influence on OHRQoL.

Upon closer analysis, it is evident that the responses in the ECOHIS questionnaire differed significantly from those in the CPQ questionnaire. While the majority (54.4%) of ECOHIS respondents reported “never” or “rarely” experiencing “tooth pain” in the affected young child, the CPQ respondents mostly (45.3%) perceived pain “occasionally” or “often”. The ECOHIS evaluation study by Pahel et al. found a more frequent rating of “never” or “rarely” in response to the pain question (83.1%) compared with our cohort [13]. Similarly, for other “most common problems” (such as “difficulty drinking hot/cold beverages”, 63% and “difficulty eating certain foods”, 50%), a majority of ECOHIS respondents in our study reported these problems as “never” occurring. This is corroborated by data from an Italian evaluation conducted in 2020 [28]. Pahel and colleagues found even more frequent positive responses such as “never” and “rarely” indicative of good OHRQoL in the populations they studied compared with our study [13].

In conclusion, the relationships between the ECOHIS domains and the “general health status” and “oral health status” should be discussed. A significant, “moderately” strong correlation was found between the “general health status” and the “family section” (ρ = 0.31; *p* = 0.03). Similarly, a significant correlation was found between the “oral health status” and the “family section” (ρ = 0.33; *p* = 0.03). Thus, poor (oral) health was associated with high response values in the family section, indicating a significant disruption in family life. No correlations were found between the “child section” and the “general sum scores” with the “general health status” and “oral health status”. This could be attributed to the small sample sizes.

In some of the studies conducted under normal conditions, the study populations were subdivided into subgroups based on diagnoses, enabling a comparison with our work. Abanto et al. differentiated Brazilian patients regarding their OHRQoL into “caries-affected” (16.65 ± 11.56), “with trauma” (8.60 ± 8.81), and “with anterior malocclusion” (8.19 ± 8.84) [18]. Another population studied in Mexico showed significantly lower values for these diagnoses [20].

For “caries-affected” individuals, the average ECOHIS sum score was 3.26 (±0.39), for “with trauma” it was 5.4 (±1.95), and for “open bite” it was 3.1 (±0.26). Bekes et al. collected an ECOHIS_Σ of 8.6 (±6.7) in the Austrian normal collective for caries-affected individuals [11]. In China, an ECOHIS_Σ of 14.98 (±6.99) was found for caries-affected individuals [19]. Comparing the caries sum scores of these studies with our work (13.77 ± 9.885), it can be observed that the results of the Brazilian (16.65 ± 11.56) and Chinese studies (14.98 ± 6.99) were closest to the values we determined [18,19].

An Austrian study of the CPQ found a total score of 7.5 [±8.6] in children without caries compared with 9.1 [±10.1] in children with caries [11]. This means that the score of those affected by caries was more than half of the one we determined (16.33 [±12.38]). At the beginning of the pandemic, caries patients in Austria perceived their MLQ (Mean Life Quality) as significantly worse than under normal conditions.

In other evaluation studies, such as those from the Arab Emirates (23.3 [±19.0]) and Cambodia (22.4 [±13.6]), the total scores of those affected by caries also reached a higher level than the caries total score we determined [29,30]. However, the scores from the previously cited Turkish (13.2 [±9.1]) and Mexican (14.87) studies were lower than our caries total score [31,32]. The impairment of the MLQ due to caries in our study was statistically in the mid-range compared with studies that considered this item under different conditions.

The average overall sum score of CPQ participants in our study was CPQ_Σ 18.36. With a maximum possible sum score of 140 points, this average indicated a low OHRQoL impairment. However, the range of 0–50 was very large, suggesting many different response values and thus different weights of questions. This was also evident from the large standard deviation of ±12.35. The sum score of 18.36 is shifted to the left in the range, confirming the relatively low average OHRQoL impairment. Upon closer examination of the individual questions, the significantly negative influence of the symptom “tooth pain” on OHRQoL was confirmed (1.75 ± 1.22). A Brazilian study published in 2021 also reached the same conclusion, where the negative effects of tooth pain were more strongly associated with poorer OHRQoL than dental malocclusions [33]. Four other questions stood out in our analysis with values greater than 1.0 compared with the rest of the items. One question was, “How often food got stuck in the teeth” (1.68 ± 1.36). It also belongs to the domain of oral symptoms and had nearly the same negative impact on OHRQoL as the question about “pain”. The other three items that contributed most to reducing OHRQoL were: how often the child “got annoyed”, experienced “pain when drinking cold beverages or eating”, or had “difficulty eating or chewing foods such as apples, corn on the cob, or meat”. These questions and related common answers give a clear indication of the influence of oral health on the quality of life.

Most participants experienced “tooth pain” “often”, making it a particularly distressing symptom. In contrast, in the domains of “pain when drinking cold beverages or eating” and “food getting stuck in the teeth”, the response values were relatively evenly distributed. The frequency of response values such as “very often”, “often”, “occasionally”, etc. varied little. Most participants reported “never” having a problem with “bad breath”. This contrasted with the results of a study published in 2011, which surveyed a group of adolescents aged 11–14 years using the CPQ-G [34]. In that study, foetor ex ore was perceived as “often” or “very often”.

The older children, like the younger ones in the ECOHIS, answered the question about “oral health status” with more negative scores than the question about “general health status”. While only 69.8% rated their “oral health status” as “good” or better, with 30.2% rating it as “fair” or “poor”, the “general health status” was rated as “good” or better by 96.2% of the cohort, with none rating it as “poor”. From the participants’ perspective, oral health thus had little influence on general health.

Analyzing the correlations of the domains with “oral health status” and “general health status”, it can be observed that none of the domains nor the overall sum score correlated significantly with “general health status”. However, there was a tendency for a relationship between oral symptoms and general health, for instance. With a larger sample size, the significance level might have been reached. Two out of the four domains correlated significantly with “oral health status”. A statistically significant, “moderate” causal relationship was observed between “oral symptoms” and “emotional well-being”. This demonstrates an interaction between “oral symptoms” and “emotional well-being” with “oral health status”. The factors in the pandemic exerting a strongly negative influence on “oral symptoms” remain speculative. It is possible that patients, due to social restrictions and regulations, had more time to focus on their personal issues. Additionally, the population’s awareness of “health changes” may have been heightened due to the confrontation with the novel, unexplored virus. Another reason for the high OHRQoL scores could have been the late presentation to emergency dental services, leading to significantly reduced oral health. Better prevention and thus avoidance of OHRQoL deterioration could be achieved in the future if patients present themselves to emergency services more promptly under pandemic restrictions. Also, regular visits to dental practices without the pressure of a crisis situation would mitigate the negative OHRQoL development. These unconfirmed speculations could serve as a research approach for further studies.

This study is the first systematic analysis of OHRQoL in Austrian children during the early COVID-19 pandemic, but it has limitations. The small study group reduces statistical significance, and the results, reflecting Vienna’s situation in the pandemic’s early weeks, may not apply to other regions.

New health strategies to combat the pandemic were implemented after the first 6 weeks of the study. Further research is needed to investigate their impact on oral health and OHRQoL.

Another study limitation is not considering the respondent’s gender in the toddler group (ECOHIS). Research shows fathers and mothers assess children’s health differently, with mothers generally providing more reliable answers about oral health [35]. Furthermore, some participants misunderstood the questionnaires, leaving notes indicating confusion and occasionally overlooking the back page, leading to unwanted exclusions. On some days, no questionnaires were answered due to pandemic-related staff shortages, while more were completed on following days. Additionally, comparing our results with other studies was impossible due to the lack of similar research. It is also worth mentioning that socioeconomic status significantly impacts healthcare in general and oral health in particular. However, our study’s questionnaires did not allow for its assessment. This topic could be a focus for future research. Emergency treatment protocols and the overall approach within the Austrian healthcare system require further investigation, strategic planning, and practical solutions for emergency units that provide daily care to patients in need. Broader multidisciplinary studies are necessary to ensure and sustain adequate patient care services.

Lastly, the outbreak of the pandemic was an unexpected event with an unpredictable course of infection. The pandemic caught both the population and dental care completely unprepared. During the observation period, new strategies had to be constantly developed to combat the disease and maintain healthcare services. At the beginning of our six-week observation, the OHRQoL may have been assessed differently than at the end, as circumstances changed rapidly. It is likely that today, over two years after the start of the pandemic, a survey would yield completely different results.

## 5. Conclusions

The early COVID-19 pandemic significantly impacted the OHRQoL of children aged 0–10 years old.

The main reasons for impaired OHRQoL were toothaches. In the younger group (ECOHIS), oral health was influenced by family circumstances, while in CPQ, it was affected by oral symptoms and emotional well-being.

## Figures and Tables

**Table 1:** Characteristics of the study population for ECOHIS and CPQ groups.

	ECOHIS Group (0–6 Years)		CPQ Group (7–10 Years)
Gender	Overall n (%)	Gender	Overall n (%)
Male ♂	20 (43)	Male ♂	33 (62)
Female ♀	26 (56)	Female ♀	20 (38)
Overall	46 (100)	Overall	53 (100)
Age		Age	
Mean	4 y	Mean	8 y
±Standard deviation	1.6	±Standard deviation	2.1
Range	0–7 y	Range	5–14 y

**Table 2:** ECOHIS—most common problems (n = 46).

ECOHIS Item	Very Often n (%)	Often n (%)	Sometimes n (%)	Rarely n (%)	Never n (%)
Toothache	4 (8.7)	8 (17.4)	9 (19.6)	12 (26.1)	13 (28.3)
Difficulty drinking hot/cold beverages	1 (2.2)	4 (8.7)	5 (10.9)	7 (15.2)	29 (63.0)
Difficulty eating certain foods	1 (2.2)	8 (17.4)	2 (4.3)	12 (26.1)	23 (50.0)
Difficulty pronouncing certain words		5 (10.9)	1 (2.2)	8 (17.4)	32 (69.6)
Missed kindergarten/daycare/school	1 (2.2)	2 (4.3)	3 (6.5)	6 (13.0)	34 (73.9)
Sleeping problems		7 (15.2)	3 (6.5)	8 (17.4)	28 (60.9)

**Table 3:** ECOHIS correlation: general/oral health status and domains or total score (n = 46).

	General Health Status		Oral Health Status	
ECOHIS Domains	Spearman’s Rank Correlation Coefficient	Significance	Spearman’s Rank Correlation Coefficient	Significance
ECOHIS Score	0.35	0.82	0.13	0.40
Children Section	0.18	0.24	0.27	0.07
Family Section	0.31 *	0.03	0.33 *	0.03

* The correlation is significant at *p* < 0.05.

**Table 4:** ECOHIS—most common diagnoses and their (domain) total scores (n = 46).

Diagnosis	Mean	±Standard Deviation	Range
Caries (31)			
Total Score	13.7	9.885	0–42
Children Section	7.68	7.54	0–30
Family Section	2.90	2.88	0–8
Dental Trauma (anterior teeth) (9)			
Total Score	7.33	3.64	0–13
Children Section	2.33	3.00	0–8
Family Section	0.22	0.67	0–2

**Table 5:** CPQ—most common oral health-related problems (n = 53).

CPQ Item	Very Often n (%)	Often n (%)	Occasional n (%)	Once/Twice n (%)	Never n (%)
Toothache	4 (7.5)	13 (24.5)	11(20.8)	16 (30.2)	9 (17.0)
Oral sore spots	-	4 (7.5)	4 (7.5)	10 (18.9)	35 (66.0)
Pain when drinking cold beverages or eating	1 (1.9)	13 (24.5)	11 (20.8)	9 (17.0)	19 (35.8)
Bad breath	1 (1.9)	6 (11.3)	5 (9.4)	6 (11.3)	35 (66.0)
Food stuck in your teeth	6 (11.3)	9 (17.0)	15 (28.3)	8 (15.1)	15 (28.3)
Taking longer than others to eat your meal	1 (1.9)	8 (15.1)	6 (11.3)	7 (13.2)	31 (58.5)

**Table 6:** CPQ correlation general/oral health status and domains or sum score (n = 53).

	General Health Status		Oral Health Status	
CPQ Domains	Spearman’s Rank Correlation Coefficient	Significance	Spearman’s Rank Correlation Coefficient	Significance
CPQ Sum Score	0.09	0.52	0.23	0.09
Oral Symptoms	0.49	0.73	0.36	0.01
Functional Limitations	0.06	0.66	0.25	0.07
Emotional Well-being	0.14	0.31	0.34	0.01
Social Well-being	0.22	0.12	0.07	0.63

**Table 7:** CPQ—most common diagnoses and their (domain) sum scores (n = 53).

Diagnosis	Mean	±Standard Deviation	Range
Caries (27)			
Sum Score	16.33	12.38	0–50
Oral Symptoms	5.85	4.25	0–15
Functional Limitations	3.41	4.12	0–12
Emotional Well-being	3.00	3.44	0–12
Social Well-being	1.04	1.87	0–8
Others * (13)			
Sum Score	20.54	12.48	8–49
Oral Symptoms	6.31	3.64	1–15
Functional Limitations	5.15	4.98	0–13
Emotional Well-being	3.77	4.21	0–15
Social Well-being	1.08	2.25	0–6

* Others = broken fillings/crowns, space maintainer fracture/pressure sore, fracture, sensitivity pain, mobility, teething pain.

## Data Availability

All data supporting the reported results are available upon request from the corresponding author.

## Abbreviations

The following abbreviations are used in this manuscript:

OHRQoL	Oral health-related quality of life
ECOHIS	Early Childhood Oral Health Impact Scale questionnaire
CPQ	Child Perception Questionnaire
QoL	Quality of life
CPQ-G	German version of the Child Perception Questionnaire
